# Effects of the interaction between cold spells and fine particulate matter on mortality risk in Xining: a case-crossover study at high altitude

**DOI:** 10.3389/fpubh.2024.1414945

**Published:** 2024-05-15

**Authors:** Zhenxu Ning, Shuzhen He, Qiansheng Liu, Haibin Ma, Chunguang Ma, Jing Wu, Yanjun Ma, Youxia Zhang

**Affiliations:** ^1^Department of Public Health, Faculty of Medicine, Qinghai University, Xining, China; ^2^Xining Centre for Disease Control and Prevention, Xining, China; ^3^Qinghai Institute of Health Sciences, Xining, China; ^4^Qinghai Province Cardio Cerebrovascular Disease Specialist Hospital, Xining, China

**Keywords:** cold spell, fine particulate matter, mortality, air pollution, climate change

## Abstract

**Background:**

With global climate change, the health impacts of cold spells and air pollution caused by PM_2.5_ are increasingly aggravated, especially in high-altitude areas, which are particularly sensitive. Exploring their interactions is crucial for public health.

**Methods:**

We collected time-series data on meteorology, air pollution, and various causes of death in Xining. This study employed a time-stratified case-crossover design and conditional logistic regression models to explore the association between cold spells, PM_2.5_ exposure, and various causes of death, and to assess their interaction. We quantitatively analyzed the interaction using the relative excess odds due to interaction (REOI), attributable proportion due to interaction (AP), and synergy index (S). Moreover, we conducted stratified analyses by average altitude, sex, age, and educational level to identify potential vulnerable groups.

**Results:**

We found significant associations between cold spells, PM_2.5_, and various causes of death, with noticeable effects on respiratory disease mortality and COPD mortality. We identified significant synergistic effects (REOI>0, AP > 0, S > 1) between cold spells and PM_2.5_ on various causes of death, which generally weakened with a stricter definition of cold spells and longer duration. It was estimated that up to 9.56% of non-accidental deaths could be attributed to concurrent exposure to cold spells and high-level PM_2.5_. High-altitude areas, males, the older adults, and individuals with lower educational levels were more sensitive. The interaction mainly varied among age groups, indicating significant impacts and a synergistic action that increased mortality risk.

**Conclusion:**

Our study found that in high-altitude areas, exposure to cold spells and PM_2.5_ significantly increased the mortality risk from specific diseases among the older adults, males, and those with lower educational levels, and there was an interaction between cold spells and PM_2.5_. The results underscore the importance of reducing these exposures to protect public health.

## 1 Introduction

In the current context of global climate change, the frequency and intensity of extreme weather events are showing an upward trend, posing significant threats to human health and society (1). Cold spells, characterized by sudden and severe drops in temperature, together with the ongoing issue of air pollution, exacerbate the risks to public health (2, 3). Several studies have revealed the association between cold environments and PM_2.5_ pollution with a range of health problems, including cardiovascular diseases, respiratory diseases, and diabetes (4, 5). In high-altitude areas, thin air, strong ultraviolet radiation, and increased human activities may have exacerbated the problem of air pollution in the environment (6–8). Studies have shown that air pollution in high-altitude regions has a significant impact on human health (9, 10). For example, a time-series analysis revealed a correlation between air pollution and respiratory health issues in children in the Xining area (11). Moreover, the impact of indoor air pollution in these areas on residents’ health is even more severe (12, 13). Research on the health impacts of cold spells and air pollution in high-altitude areas is relatively scarce due to their unique geographical and climatic conditions and the specificity of population distribution, limiting our comprehensive understanding of the health impacts of extreme climate and air pollution in these regions. Additionally, the low oxygen and low pressure environment of high-altitude areas may negatively impact health through various physiological mechanisms, such as triggering the activation of hypoxia-inducible factors, enhancing inflammatory responses, and damaging mitochondrial function (14–17). Under extreme weather conditions, such as cold spells, these health effects may be further amplified. Therefore, in high-altitude areas, it is crucial to fully elucidate the correlation between cold spells, air pollution, and health outcomes for a thorough understanding and targeted prevention and adaptation.

Several studies have shown that the combination of extreme weather events and air pollution can have negative effects on human health (18, 19). However, most of these studies have focused on the impact of extreme high temperatures, with less attention given to the potential health impacts of different intensities, frequencies, and durations of extreme weather events such as cold spells. Furthermore, these studies assess the interaction between the two factors by calculating the relative excess risk due to interaction (RERI), but this single indicator does not take into account the possible complex synergistic effects between them. Xining City, being a high-altitude area, may exacerbate the negative health effects of air pollution due to its unique geographical and climatic conditions. The city experiences a six-month heating period during the winter and longer cold periods. This scenario highlights the need for a more comprehensive and detailed assessment method when considering the combined effects of extreme weather events and air pollution in high-altitude areas, in order to fully understand and evaluate the potential health impacts of these environmental factors’ synergistic effects.

To complement existing research, this study aims to quantitatively assess the interactive effects of cold spells and PM_2.5_ exposure on the risk of various causes of death in high-altitude areas and to calculate the corresponding excess mortality rates and numbers. In addition, this study will conduct stratified analyses to identify potentially vulnerable groups, in particular through stratification by average altitude, to further explore the effects of cold spells in high-altitude areas.

## 2 Materials and methods

### 2.1 Data collection and definition of cold spell

We collected mortality data from January 1, 2016, to December 31, 2021, from the Xining City Center for Disease Control and Prevention, including age, sex, level of education, disease diagnosis, and codes according to the International Classification of Diseases, Tenth Revision (ICD-10). The data were categorized by average altitude (2,500 m and 3,000 m), sex (male and female), age (0–64 years and ≥ 65 years), level of education (junior high school and below, high school and above), and specific causes of death: non-accidental (ICD-10:A00-R99), cardiovascular disease (ICD-10:I00-I99), ischemic heart disease (IHD, ICD-10:I20-I25), stroke (ICD-10:I60-I69), respiratory disease (ICD-10:J00-J99), chronic obstructive pulmonary disease (COPD, ICD-10:J40-J47), and diabetes (ICD-10:E10-E14). For altitude classification, we referred to the categories proposed by Bärtsch et al. in 2008 (near sea level 0–500 meters, low altitude 500–2,000 meters, mid altitude 2000–3,000 meters, high altitude 3,000–5,500 meters, extreme altitude above 5,500 meters) (20, 21). Meteorological data were obtained from the Qinghai Provincial Meteorological Bureau, including daily average temperature and humidity, with no missing data. Air pollutant data were sourced from the China Air Quality Online Monitoring and Analysis Platform,^1^ supplemented by five national control monitoring stations in the urban area of Xining. The overall missing rate of air pollutant data was about 1.76%, including daily average concentrations of PM_2.5_, PM_10_, O_3_, SO_2_, NO_2_, and CO. For missing air pollution data, we used the median imputation method for data filling. We used Spearman’s correlation analysis of meteorological factors and pollutants.

Based on previous studies, cold spells were defined as daily average temperatures falling below specific percentiles (2.5, 5, 7.5, or 10th) and persisting for a minimum of 2 to 4 consecutive days (22, 23).

### 2.2 Study sites

**Figure 1:**
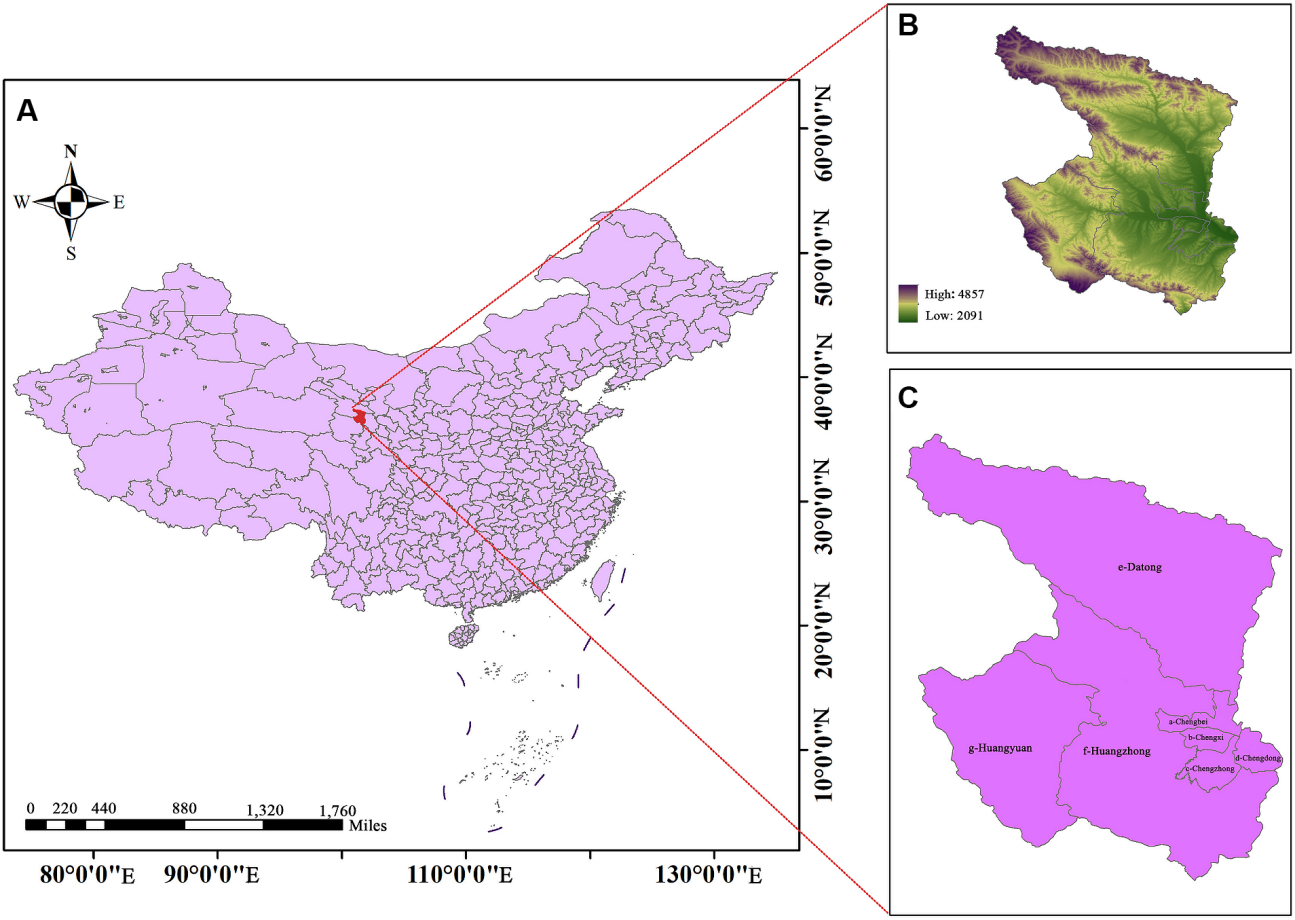
Location, altitude range and topography of Xining, China (**C**: a is Chengbei District, b is Chengxi District, c is Chengzhong District, d is Chengdong District, e is Datong County, f is Huangzhong District, and g is Huangyuan County). The average altitude of a-d is about 2500 m, and the average altitude of e-g is about 3000 m.

Xining City is located in the northwest of China and the northeast of the Tibetan Plateau, with an altitude range of 2091–4,857 meters, making it one of the world’s high-altitude cities. The terrain is higher in the southwest and lower in the northeast, with a total population of about 2.4756 million people (in 2021), accounting for approximately 42% of Qinghai Province (Figure 1). The climate is characterized by a high mountain plateau climate, with cold and prolonged winters and pleasant summers.

### 2.3 Statistical analysis

We used a time-stratified case-crossover design and conditional logistic regression to quantitatively analyze the association between cold spells, PM_2.5_, and mortality (24). The case-crossover design considers each study subject as its own control, with the visit date defined as the case day, and other dates in the same year, month, and week as the case day defined as control days. Each case period is matched with three or four control periods before or after the case period to control for long-term trends, seasonal trends, and the effects of the day of the week.

A distributed lag non-linear model (DLNM) was applied to fit separate exposure-response and lag-response relationships for cold spells, PM_2.5_, and mortality (25). Based on previous studies, the exposure-response relationship was modeled using a linear function (26), while the lag-response relationship was fitted using a natural spline function with 3 degrees of freedom (df) (23). Previous studies have shown that the lag effect for cold spells was typically 21 days (4), while for PM_2.5_ it was 7 days (27). A natural spline with 3 df was used to control for the confounding effects of relative humidity (28). The formula was as follows:


$$\log\left(E\left(Y\right)\right)=\alpha +cb\left(CS_{i}/pm_{i},\mathrm{lag}\right)+\mathrm{ns}\left(rh,3\right)+\mathrm{stratum}+\mathrm{holiday}$$

where *E(Y)* is the expected daily number of deaths; *α* is the intercept; *cb(CS), cb(pm)* are the cross-basis functions for cold spells and PM_2.5_, respectively, used to examine lag effects; *stratum* is the time stratification variable, used to control for the impact of time factors such as long-term trends and seasonal changes; *ns(rh, 3)* is the natural cubic spline for relative humidity with 3 df; *holiday* is a binary variable used to control for Chinese holidays.

To further evaluate the interactive effects of exposure to cold spells and PM_2.5_ on mortality, we classified PM_2.5_ exposure into a binary variable (low concentration: ≤37.5 μg/m^3^, high concentration: >37.5 μg/m^3^) according to the interim target 3 for PM_2.5_ in the World Health Organization’s 2021 air quality guidelines (29, 30). We created a new variable with four levels representing the combinations of exposure to cold spells and PM_2.5_, including: (1) non-cold spell and low-level PM_2.5_ (Level 1); (2) cold spell and low-level PM_2.5_ (Level 2); (3) non-cold spell and high-level PM_2.5_ (Level 3); (4) cold spell and high-level PM_2.5_ (Level 4), with Level 1 serving as the reference group. By incorporating this variable into the conditional logistic regression model, we used three measures to assess this impact, including the relative excess odds due to interaction (REOI), the proportion attributable to interaction (AP), and the synergy index (S), which represents the part of the effect due to interaction (31, 32). The proportion of the joint effect due to interaction, as well as the ratio of the joint effect to the independent effects, were calculated using the following formulas:


$$\mathrm{REOI}=\left(OR_{11}-1\right)-\left(OR_{10}-1\right)-\left(OR_{01}-1\right)=OR_{11}-OR_{10}-OR_{01}+1$$

$$AP=\frac{\mathrm{REOI}}{OR_{11}}$$

$$S=\frac{OR_{11}-1}{\left(OR_{10}-1\right)+\left(OR_{01}--1\right)}$$

where OR_10_, OR_01_, OR_11_ are the OR values of Levels 2, 3, and 4 relative to Level 1 (OR_00_ = 1), respectively. REOI = 0, AP = 0, S = 1 indicates that there is no interaction between cold spells and PM_2.5_ on mortality; REOI>0, AP > 0, S > 1 indicates that the combined effect of cold spells and PM_2.5_ on mortality is greater than the sum of the effects of exposure alone (synergistic effect); whereas REOI<0, AP < 0, S < 1 indicates that the combined effect is less than the sum of the effects of each exposure alone. The 95% confidence intervals (CI) for the three indicators are calculated using the delta method (33).

To estimate the excess mortality attributable to simultaneous exposure to cold spells and high levels of PM_2.5_, this study used the calculation method [exp(*β*)-1], where *β* represents the coefficient of the fourth level of exposure in the conditional logistic regression model (34).

To identify potential vulnerable groups, we classified them by altitude, sex, age, and level of education to assess the independent impacts of cold spells and PM_2.5_ on non-accidental deaths and their interaction. We used a two-sample z-test to examine the differences in the effects estimated for each stratified variable (35–37).


$$Z=\frac{\beta_{2500m}-\beta_{3000m}}{\sqrt{SE_{2500m}^{2}-SE_{3000m}^{2}}}$$

where *β* represents the specific point estimate in the conditional logistic regression model; *SE* represents the standard error corresponding to each *β*.

### 2.4 Sensitivity analysis

Several sensitivity analyses were performed to test the robustness of our results. The lag days for cold spells were adjusted from 0–21 days to 0–27 days, and for PM_2.5_ from 0–7 days to 0–10 days, while the degrees of freedom for relative humidity in the model were adjusted from 3 to 6. Simultaneously, we incorporated individual air pollutants (NO_2_, CO, SO_2_, and O_3_) and a combination of air pollutants (NO_2_ & SO_2_ & CO) into the model. A new variable of different levels was created using the value of 39.5 μg/m^3^ for PM_2.5_ categorization to observe changes in the synergistic effect. Moreover, to observe potential disturbances brought by the COVID-19 pandemic, we divided the study data into two periods: 2016–2019 as the pre-pandemic control period and 2020–2021 as the pandemic period. This study primarily utilized R software for statistical analysis (version 4.3.1). *p* < 0.05 (two-sided) were considered statistically significant.

## 3 Results

**Figure 2:**
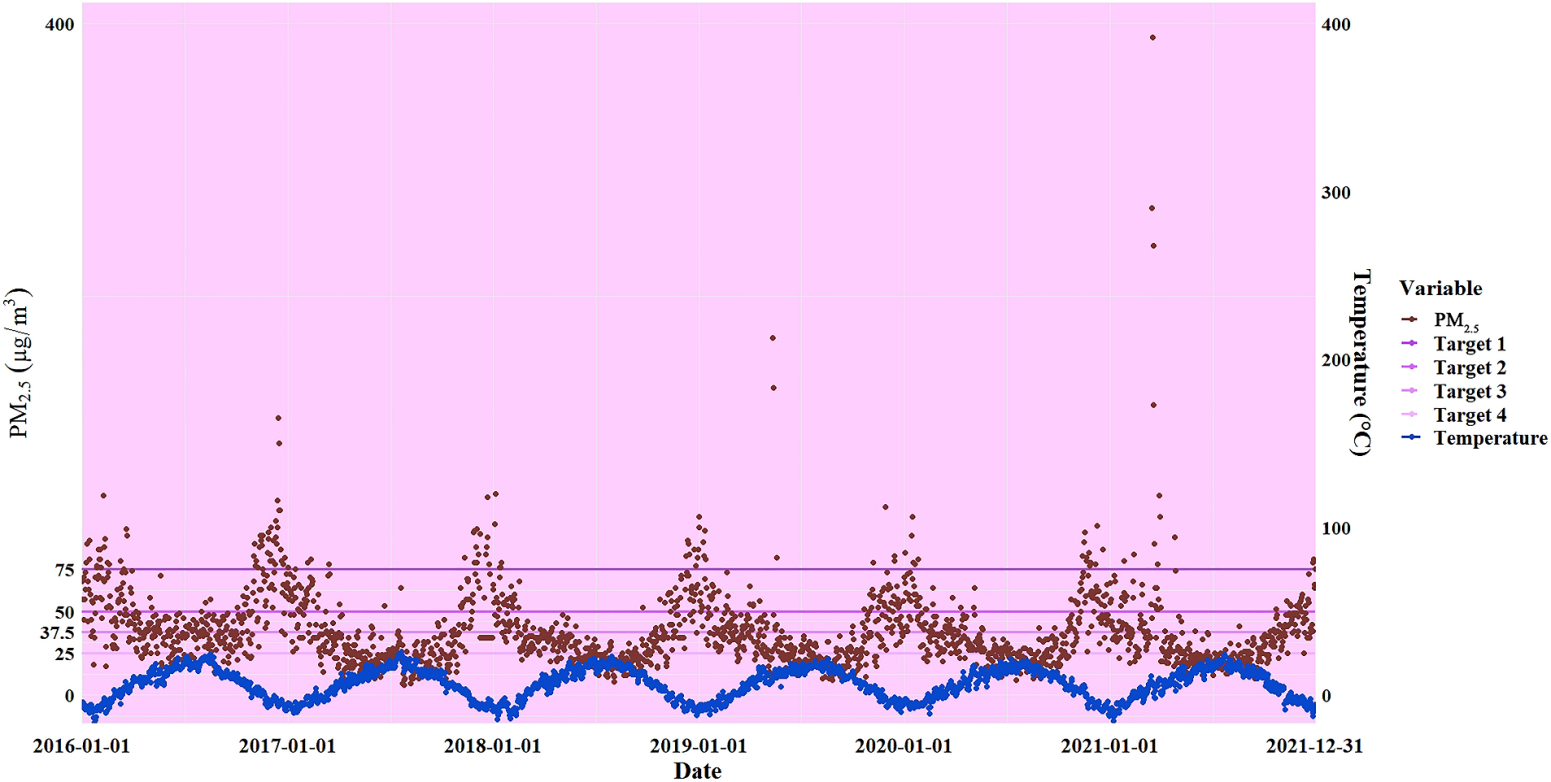
Trends in temperature and PM_2.5_ over time and interim targets for daily average PM_2.5_ concentrations according to the World Health Organization’s 2021 Air Quality Guidelines (interim target 4 is 25 μg/m^3^, 3 is 37.5 μg/m^3^, 2 is 50 μg/m^3^, 4 is 75 μg/m^3^, and HIGH is greater than 75 μg/m^3^).

Supplementary Table S1 presents a descriptive analysis of meteorological elements, atmospheric pollutants, and the number of specific causes of death. During the study period, the average daily temperature and relative humidity in Xining City were 6.46 ± 9.15 (°C) and 56.69 ± 16.20 (%), respectively. The daily average concentrations of PM_2.5_, SO_2_, NO_2_, CO, and O_3_ were 40.20 ± 27.90 μg/m^3^, 20.03 ± 13.12 μg/m^3^, 39.31 ± 16.11 μg/m^3^, 1.36 ± 0.78 mg/m^3^, and 93.31 ± 33.47 μg/m^3^, respectively. From 2016 to 2021, there were a total of 64,128 non-accidental deaths, 29,906 deaths from circulatory disease, 8,553 from respiratory disease, 11,617 from IHD, 12,237 from stroke, 7,074 from COPD, and 2,560 from diabetes. Figure 2 reveals the trend of PM_2.5_ versus temperature over time, showing the temporal synchronization of high PM_2.5_ levels with the occurrence of low temperatures. A low to moderate correlation existed between the average daily temperature and other variables (*p* < 0.05; Supplementary Figure S1). Among these variables, the correlation between PM_2.5_ and PM_10_ was relatively strong (*p* < 0.05), with a correlation coefficient greater than 0.8. However, the correlation between O_3_ and relative humidity was minimal (*p* > 0.05).

Table 1 shows the number of non-accidental deaths at different exposure levels in Xining City from 2016 to 2021. According to the definition of a cold spell by 7th4D, there were 142 cold spell days, with 4,102 cases (6.4%) of deaths occurring on cold spell days, and the majority of deaths (60,026 cases) occurred on non-cold spell days. Among these, 87.9% (3,605) of the deaths occurred under the condition of simultaneous exposure to cold spells and high levels of PM_2.5_, while 12.1% (497) occurred under simultaneous exposure to cold spells and low levels of PM_2.5_. Overall, the number of non-accidental deaths during cold spell days decreased with lower temperature thresholds and longer durations.

**Table 1:** Number of non-accidental deaths during cold spell events in Xining, China, 2016–2021.

Definition	Days	Deaths (%)		
		Overall	With low-level PM_2.5_	With high-level PM_2.5_
10th2D	201	6,976	10,11(14.5)	5,965(85.5)
10th3D	182	6,288	923(14.7)	5,365(85.3)
10th4D	164	5,647	685(12.1)	4,962(87.9)
7.5th2D	163	5,703	869(15.2)	4,834(84.8)
7.5th3D	142	4,994	664(13.3)	4,330(86.7)
7.5th4D	119	4,102	497(12.1)	3,605(87.9)
5th2D	98	3,398	428(12.6)	2,970(87.4)
5th3D	81	2,809	396(14.1)	2,413(85.9)
5th4D	63	2,209	311(14.1)	1,898(85.9)
2.5th2D	46	1,530	287(18.8)	1,243(81.2)
2.5th3D	40	1,342	287(21.4)	1,055(78.6)
2.5th4D	31	1,142	233(20.4)	909(79.6)

**Figure 3:**
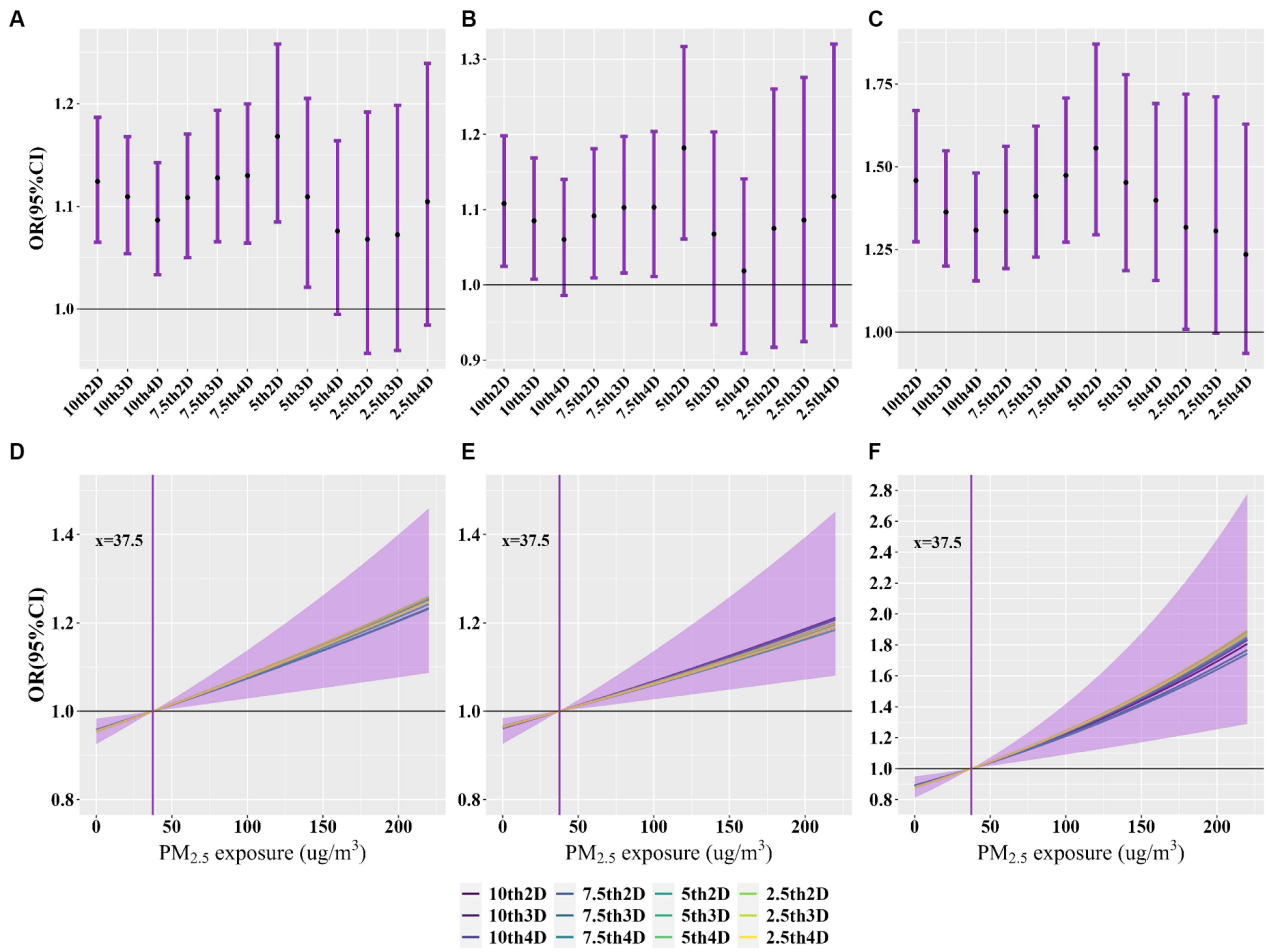
Association of exposure to cold spells and PM_2.5_ with non-accidental, cardiovascular and respiratory disease deaths. **(A**–**C)** OR (95%CI) for nonaccidental, cardiovascular, and respiratory disease deaths with exposure to cold spells, respectively. **(D–F)** Exposure-response curves for the association between exposure to ambient PM_2.5_ and non-accidental, cardiovascular and respiratory disease deaths, respectively.

Figures 3A–C and Supplementary Figures S2A–D show the relationship between exposure to cold spells and various causes of death. We observed a significant increase in the risk of death from all causes associated with exposure to cold spells. According to the 5th2D definition of cold spells, the odds ratios (OR) for total non-accidental deaths, deaths from circulatory disease, and respiratory disease were 1.168 (95%CI: 1.085, 1.258), 1.182 (1.061, 1.317), and 1.556 (1.294, 1.871), respectively, indicating an increase in death risk of 16.8% (8.5, 25.8%), 18.2% (6.1, 31.7%), and 55.6% (29.4, 87.1%). Overall, the risk of death decreased with stricter definitions of cold spells, and the confidence intervals of effect estimates became wider, with a higher risk of death from respiratory disease.

Figures 3D–F and Supplementary Figures S2E–H show the association between exposure to PM_2.5_ and various causes of death. After adjusting for different definitions of cold spells in the model, the OR for total non-accidental deaths, deaths from circulatory disease, and respiratory disease monotonically increased with higher exposure to PM_2.5_, with the highest risk for respiratory disease.

**Figure 4:**
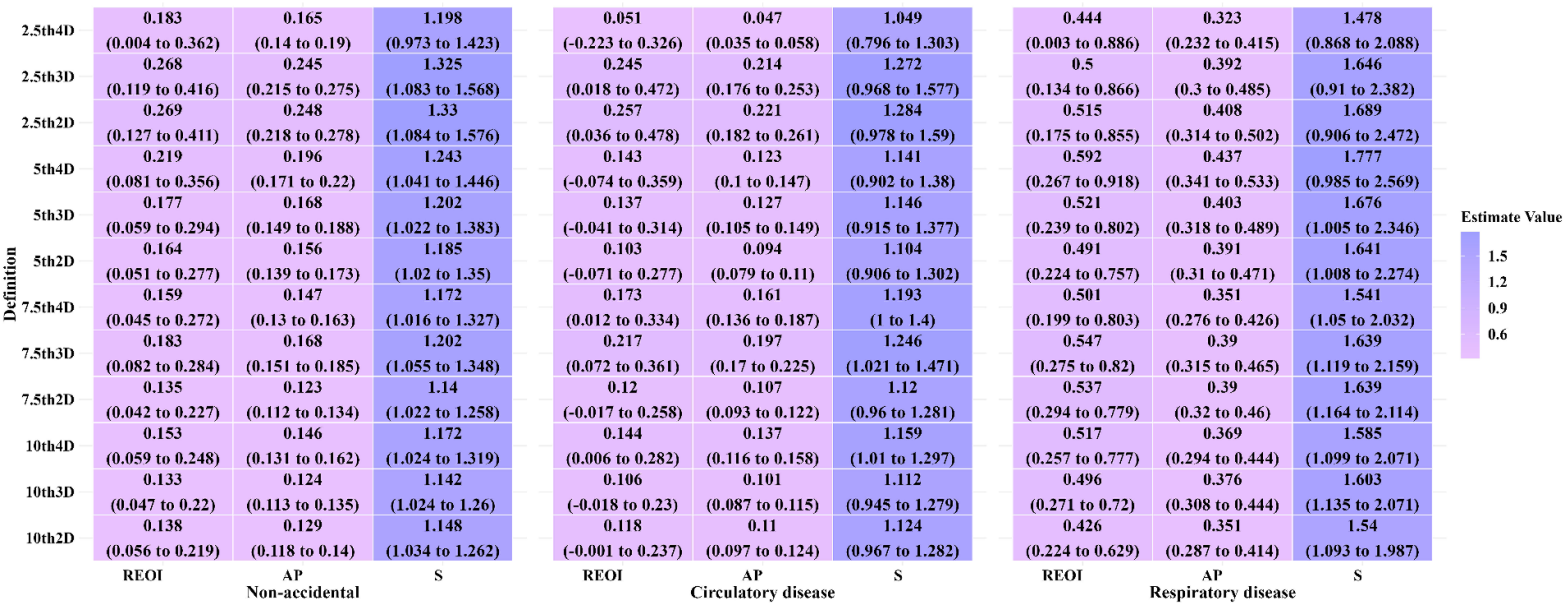
Additive interaction effects of exposure to cold periods and PM_2.5_ on non-accidental, cardiovascular and respiratory disease mortality.

Figure 4 and Supplementary Figure S3 show the additive interactive effects on specific causes of death due to exposure to cold spells and PM_2.5_. According to the 7th4D definition of cold spells, the REOI for total non-accidental deaths, deaths from circulatory disease, respiratory disease, IHD, stroke, COPD, and diabetes were 0.159 (95%CI: 0.045, 0.272), 0.173 (0.012, 0.334), 0.501 (0.199, 0.802), 0.353 (0.077, 0.628), 0.431 (0.189, 0.673), 0.354 (0.009, 0.699), and 1.109 (0.621, 1.598), respectively. Except for IHD, COPD, and diabetes, other causes indicated a significant synergistic effect of exposure to cold spells and PM_2.5_ on mortality (indicated by REOI >0, AP >0, and S > 1; all *p* < 0.05). Overall, the REOI, AP, and S decreased with lower temperature thresholds and longer durations of cold spells, and the confidence intervals also became wider.

**Figure 5:**
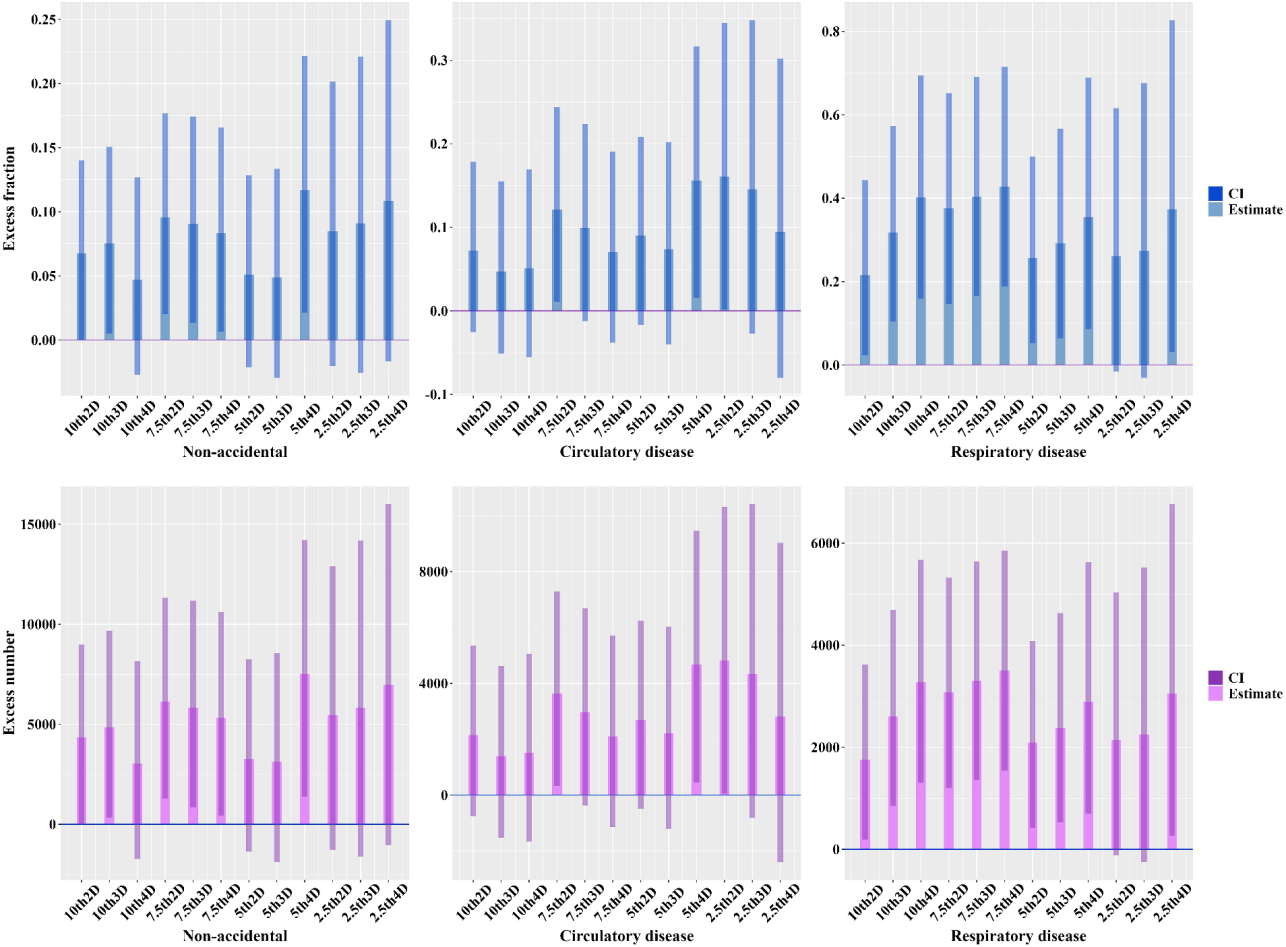
Excess fraction and number of excess deaths due to exposure to cold spells and high-level PM_2.5_.

Figure 5 and Supplementary Figures S4, S5 show the excess fraction and number of excess deaths due to exposure to cold spells and high-levels of PM_2.5_. According to the 7th3D definition of cold spells, the excess mortality rates for total non-accidental deaths, deaths from circulatory disease, respiratory disease, IHD, stroke, COPD, and diabetes were 0.083 (95%CI: 0.007, 0.166), 0.070 (−0.038, 0.191), 0.428 (0.188, 0.716), 0.330 (0.129, 0.567), 0.216 (0.033, 0.430), 0.421 (0.183, 0.708), and 0.607 (0.179, 1.191), respectively. Overall, significant findings were mainly concentrated between 7th2D to 7th4D, with lower excess mortality rates associated with lower temperature thresholds and longer durations of cold spells.

Table 2 and Supplementary Figures S6, S7 show the OR and additive interactive effects for non-accidental deaths under different definitions of cold spells, stratified by average altitude, sex, age, and education level. We observed that, in the independent effects of exposure to cold spells on non-accidental deaths, the effect was slightly higher in populations living at an average altitude of 3,000 m compared to those at 2,500 m, slightly higher in males compared to females, lower in the 0–64 age group compared to those aged ≥65, and higher in individuals with lower educational levels compared to those with higher educational levels; however, no statistically significant differences were observed between them (*p* > 0.05; Supplementary Figure S6). In the independent effects of exposure to PM_2.5_ on non-accidental deaths, the effect was slightly lower in populations living at an average altitude of 3,000 m compared to those at 2,500 m, similar between males and females, lower in the 0–64 age group compared to those aged ≥65, and higher in individuals with lower educational levels compared to those with higher educational levels, but no significant differences were observed across the groups (*p* > 0.05; Supplementary Figure S7). We observed a significant synergistic effect (*p* < 0.05; Table 2) in the interaction between exposure to cold spells and PM_2.5_ on non-accidental deaths for the age group ≥65 compared with the age group 0–64 in the 7th3D and 7th4D definitions, indicating a significant synergistic effect. No significant differences in the synergistic effect (meeting the conditions of REOI >0, AP >0, and S > 1) were observed between average altitude, sex, or education level (*p* > 0.05; Supplementary Tables S2, S4).

**Table 2:** The REOI, AP and S of exposure to cold spells and PM_2.5_ on non-accidental mortality, by average altitude, sex, age and education level.

Definition	Altitude		Sex		Age (years)		Educational level	
	2,500 m	3,000 m	Male	Female	0–64	≥65	Low	High
**REOI**								
7.5th2D	0.226 (0.095 to 0.357)^a^	0.045 (−0.086 to 0.177)	0.126 (0.005 to 0.247)	0.145 (0.002 to 0.289)	0.013 (−0.158 to 0.184)	0.181(0.071 to 0.291)^a^	0.160 (0.060 to 0.259)^a^	−0.023 (−0.274 to 0.227)
7.5th3D	0.296 (0.155 to 0.437)^a^	0.071 (−0.076 to 0.217)	0.188 (0.054 to 0.322)^a^	0.176 (0.022 to 0.33)	−0.024 (−0.221 to 0.172)^*^	0.259 (0.140 to 0.378)^a^*	0.218 (0.111 to 0.325)^a^	−0.054 (−0.357 to 0.249)
7.5th4D	0.268 (0.114 to 0.422)^a^	0.044 (−0.125 to 0.214)	0.185 (0.036 to 0.335)	0.123 (−0.052 to 0.298)	−0.159 (−0.405 to 0.086)*	0.267 (0.139 to 0.396)^a^*	0.198 (0.077 to 0.319)^a^	−0.099 (−0.437 to 0.238)
**AP**								
7.5th2D	0.196 (0.172 to 0.22)^a^	0.043 (0.037 to 0.049)	0.121 (0.105 to 0.136)	0.125 (0.108 to 0.142)	0.014 (0.011 to 0.016)	0.158 (0.141 to 0.174)^a^	0.144 (0.13 to 0.158)^a^	−0.024 (−0.030 to −0.017)
7.5th3D	0.258 (0.225 to 0.291)^a^	0.068 (0.058 to 0.078)	0.176 (0.153 to 0.199)^a^	0.157 (0.134 to 0.180)	−0.026 (−0.032 to −0.020)*	0.227 (0.202 to 0.251)^a^*	0.199 (0.178 to 0.22)^a^	−0.051 (−0.068 to −0.035)
7.5th4D	0.241 (0.206 to 0.275)^a^	0.042 (0.035 to 0.049)	0.174 (0.148 to 0.199)	0.112 (0.093 to 0.130)	−0.164 (−0.209 to −0.120)*	0.238 (0.210 to 0.266)^a^*	0.181 (0.160 to 0.202)^a^	−0.100 (−0.136 to −0.063)
**S**								
7.5th2D	1.244 (1.059 to 1.429)^a^	1.045 (0.895 to 1.196)	1.137 (0.973 to 1.302)	1.143 (0.976 to 1.31)	1.014 (0.798 to 1.230)	1.187 (1.047 to 1.328)^a^	1.168 (1.037 to 1.299)^a^	0.977 (0.712 to 1.242)
7.5th3D	1.348 (1.104 to 1.592)^a^	1.073 (0.896 to 1.250)	1.214 (1.010 to 1.417)^a^	1.186 (0.976 to 1.396)	0.975 (0.752 to 1.198)*	1.293 (1.105 to 1.480)^a^*	1.248 (1.079 to 1.418)^a^	0.951 (0.684 to 1.218)
7.5th4D	1.317 (1.049 to 1.585)^a^	1.044 (0.861 to 1.226)	1.210 (0.984 to 1.436)	1.126 (0.914 to 1.337)	0.859 (0.668 to 1.050)*	1.313 (1.094 to 1.531)^a^*	1.221 (1.041 to 1.401)^a^	0.909 (0.625 to 1.194)

^*^p < 0.05, estimated using the 2-sample z test.

a Indicates that cold spells and PM_2.5_ have additive interaction.

By adjusting the lag days for cold spells from 0–21 days to 0–27 days, the lag days for PM_2.5_ from 0–7 days to 0–10 days, modifying the degrees of freedom for relative humidity in the model, and including both individual air pollutants (SO_2_, NO_2_, CO,and O_3_) and combined air pollutants (NO_2_&SO_2_&CO) in a sensitivity analysis, the estimated values for both independent and interactive effects were similar, showing minor variations (Supplementary Figures S8–S10). When using a PM_2.5_ categorization value of 39.5 μg/m^3^, the association between cold spells and PM_2.5_ with non-accidental deaths was stable (Supplementary Figures S11, S12), but the estimated value for the interaction effect between exposure to cold spells and PM_2.5_ on non-accidental deaths slightly decreased. Furthermore, the study results from different periods suggested that, during 2020–2021, the individual effects of cold spells and PM_2.5_ slightly decreased compared to previous years (Supplementary Figure S13), with the COVID-19 pandemic potentially acting as a confounding factor but also possibly related to the reduced time span and global warming. This indicated that the impact of large-scale public health events on assessing the health effects of environmental factors needs to be considered.

## 4 Discussion

We studied the association between cold spells and PM_2.5_ with mortality in high-altitude areas (Xining) and quantified their interaction. In this case-crossover study, we found a significant correlation between exposure to cold spells and PM_2.5_ and an increase in various causes of death. Cold spells could synergistically interact with PM_2.5_ to increase the risk of death, especially the risk of respiratory disease and COPD deaths. The individual effects of cold spells and PM_2.5_, as well as their interaction on various causes of death, decreased with lower temperature thresholds and longer durations. Populations that lived at an altitude of 3,000 m, males, the older adults, and those with lower educational levels seemed to be more susceptible to cold spells. The interaction between cold spells and PM_2.5_ mainly varied across age groups (*p* < 0.05).

This study has identified a significant correlation between cold spells and various causes of death, providing an important supplement to the field of research on the impact of extreme climate conditions on human health. This study found results similar to those in Ningbo (OR 95%CI: 1.156, 1.095–1.221), Tianjin (1.26:1.15–1.39), and Jinan (1.08:1.06–1.11) regarding non-accidental deaths (22, 38, 39). This finding not only confirms the consistency of the increased risk of non-accidental deaths due to cold spells but also highlights the importance of conducting research in different geographical locations. Cold spells significantly impact public health in high-altitude areas. Research data indicate that during the cold period, there is a noticeable increase in the mortality risk in highland areas such as Tibet, Xining, and Yuxi (40–42). For instance, a study focusing on provincial capitals in China shows that during cold waves, the mortality risk in Xining is higher than in other western cities and similar latitude plain areas (22). In Tibet, studies also demonstrate that as temperatures continue to drop, the risk of death increases (42). These findings emphasize that in high-altitude regions, geographic and climatic characteristics play a crucial role in assessing health risks, which is particularly important for developing effective response strategies. In terms of specific disease categories, the risk of death from circulatory disease in this study was close to that in Jinan (1.06:1.03–1.10) (38). For the risk of respiratory disease, this study was close to those in Ningbo (1.444: 1.173–1.777) and Nanjing (1.54:1.16–2.04) (39, 43). These findings further emphasize the importance of protecting the cardiovascular and respiratory systems during cold spell events. Studies in southern China (such as Shenzhen, Guangzhou, and Wuhan) show that the risk of non-accidental deaths, cardiovascular disease, and respiratory disease due to cold spells was higher than the findings of this study (22, 44, 45), which could be related to different climate adaptabilities and cold adaptabilities of populations across regions, highlighting the role of environmental conditions in affecting the health effects of cold spells (46–48). Xining, being exposed to lower temperatures for longer periods, has a stronger cold adaptation. At the same time, significant differences between indoor and outdoor temperatures may exacerbate the risk of death from circulatory and respiratory disease (49, 50). Notably, the heating period in Xining lasts up to half a year, which may further amplify the impact of temperature differences on diseases. Additionally, the heterogeneity of this study’s results may stem from inconsistencies in the definition of cold spells, the diversity of research methods, and population size differences. In terms of the risk of diabetes mortality, this study’s results were higher than those in Harbin (1.223:1.054–1.418), Chongqing (1.201:1.006–1.434), and Korea (2.02: 1.37–2.99) (51, 52), which may be related to a higher comorbidity rate of diabetes patients in this region and other chronic diseases such as hypertension, cerebrovascular disease, kidney disease, etc.

This study reveals that cold spells significantly increase the risk of circulatory and respiratory disease, associated with a series of physiological changes triggered by cold environments. Conditions of cold increase platelet and red blood cell counts, blood viscosity, and arterial pressure, collectively promoting thrombosis (53), and increasing the risk of heart attacks or strokes. Additionally, peripheral vasoconstriction leads to elevated blood pressure (54), further increasing the probability of cardiovascular diseases (55), such as hypertension (56). Regarding the respiratory system, cold environments may increase the number of granulocytes and macrophages in the respiratory tract (57), causing the respiratory mucosa to produce more mucus (58), leading to airway obstruction and inflammation (59). Additionally, it can cause reflexive constriction of the airways (60), increasing the risk of asthma and chronic cough. Furthermore, the cold affects the local immune defense of the respiratory tract (61), increasing the possibility of respiratory infections (62), such as pneumonia. This study further confirms the widespread consensus on the significant association between PM_2.5_ and various causes of death (63, 64). Numerous experimental studies show that PM_2.5_ can increase the risk of cardiovascular diseases by triggering inflammatory responses and oxidative stress (65), affecting vascular function, and promoting arteriosclerosis (66), thereby causing blood pressure to rise and cardiovascular events (67). Inhaling PM_2.5_ also acts directly on the respiratory tract, inducing inflammation and airway allergic reactions, exacerbating diseases like asthma and COPD (65, 68). These findings highlight the importance of reducing PM_2.5_ exposure to lower the risk of related diseases.

Despite increasing studies focusing on the synergistic effects of extreme weather events and PM_2.5_ on human health, a comprehensive assessment of the synergistic effects of cold spells and PM_2.5_ on specific causes of death is still lacking. This study, for the first time, uses the REOI, AP, and S to comprehensively assess the health impacts of cold spells and PM_2.5_ interactions of varying intensities and durations, providing a new perspective for research in this field. This study confirms the synergistic effect of combined exposure to cold spells and PM_2.5_ on specific causes of death, especially in increasing the risk of respiratory deaths, aligning with previous research results from the Shanghai region (69). As the intensity and duration of cold spells increased, the independent impact of the cold spells and their interaction with PM_2.5_ on health appeared to diminish. This phenomenon is partly due to the strict definition of cold waves, which not only reduces the frequency of such events but also integrates with a more efficient early warning system. Therefore, the public is able to receive alerts in a timely manner and take corresponding preventive measures, such as enhancing warmth and limiting outdoor activities, effectively reducing health risks. However, some studies have not observed significant interactions between cold spells and PM_2.5_ (19), even suggesting a possible antagonistic effect between the two (18, 70). These differences may be due to geographical variations, including climate characteristics, residents’ adaptability, and differences in assessment methods. Other factors such as socioeconomic status, individual health conditions, and regional public health policies may also influence the results. Additionally, this study reveals that combined exposure to cold spells and high levels of PM_2.5_ can lead to an excess mortality rate of up to 9.56% (95%CI: 2.02, 17.66%; non-accidental deaths). Therefore, optimizing early warning services for extreme weather events by reducing PM_2.5_ exposure can significantly enhance public health protection. This conclusion highlights the importance of integrating air quality management when developing strategies to respond to extreme weather events and taking corresponding measures to reduce PM_2.5_ exposure to mitigate its health impacts.

Our stratified analysis shows that the impact of cold spells varies among different populations, with those living at an average altitude of 3,000 m, males, the older adults, and individuals with lower educational levels being more sensitive. Populations residing at altitudes above 3,000 meters appear to be more sensitive to cold spells, possibly due to the combined effects of low temperatures and low oxygen environments at high altitudes on human physiological mechanisms (71, 72). These conditions may lead to increased blood pressure, and chronic residence can also cause chronic mountain sickness and endothelial dysfunction of the cardiovascular system (73). With increased altitude, the health risks individuals face also increase, including the risks of cardiovascular and respiratory disease (17, 74). From a social perspective, due to economic constraints, regions above 3,000 meters have insufficient heating and insulation facilities compared to lower altitudes. Conversely, the city center, at an altitude of 2,500 meters and with more frequent economic activities, faces the main challenge of higher PM_2.5_ pollution. This also demonstrates the environmental challenges faced by different areas. In cold environments, males show more sensitive physiological responses than females, especially in terms of elevated blood pressure, cardiac responses, and metabolic reactions (75, 76). Meanwhile, short-term cold exposure increases central aortic pressure and cardiac load, further increasing cardiovascular health risks (77). Additionally, males are more likely to engage in outdoor work, experience greater indoor and outdoor temperature differences, and have smoking habits, all of which may diminish their adaptability to extreme environments. With the degradation of physiological processes and the immune system (78, 79), the older adults are more susceptible to extreme weather events and atmospheric pollutants, potentially leading to a higher incidence of pre-existing conditions. Individuals with lower educational levels are more susceptible to cold spells and PM_2.5_, possibly related to factors such as low income, household sanitation conditions, insufficient heating facilities, shallow protective awareness, and demographic structure.

Limitations existed in this study. Meteorological and pollution data came from monitoring stations, not individual exposure data, which might have contributed to exposure inaccuracies. Our meteorological and PM_2.5_ data were outdoor data, and most individuals spent more time indoors in cold weather, ignoring indoor-outdoor temperature differences. Third, the study area was high-altitude, had a peculiar environment, low average temperatures, and a tiny population, limiting generalization.

## 5 Conclusion

We discovered that cold spells and PM_2.5_ increased particular disease deaths, especially in people who resided at an average altitude above 3,000 meters, males, the older adults, and those with lesser education. Cold spells could synergistically interact with PM_2.5_ to increase the risk of death. Increased cold spell intensity and duration reduced the independent and interaction effects of cold spells and PM_2.5_ on several causes of death. Our study shows that reducing cold spells and PM_2.5_ in high-altitude areas can safeguard public health, with major public health consequences.

## Data availability statement

The data analyzed in this study is subject to the following licenses/restrictions: the mortality data in this paper cannot be published as they contain a great deal of personal information about the deceased and their families. However, they can be obtained from the corresponding author on reasonable request. Requests to access these datasets should be directed to SH, hehe3991391@126.com.

## Ethics statement

The research protocol for this study has been approved by the Ethics Review Committee of the Xining Center for Disease Control and Prevention in Qinghai Province, China (Approval No.: qhxncdcllsc-2024004). All procedures were conducted in accordance with relevant guidelines and regulations. Daily death data were summarized at the city level, were retrospective, and were low-risk studies. All personal information involved in this article was conducted under the supervision of the relevant researchers at the Xining Municipal Center for Disease Control and Prevention, and only secondary aggregated data were used in the analysis, which did not involve participants’ names, identifying information, telephone numbers, or residential addresses; therefore, the Ethics Review Committee of the Xining Center for Disease Control and Prevention waived written informed consent.

## Author contributions

ZN: Conceptualization, Formal analysis, Investigation, Methodology, Software, Visualization, Writing – original draft. SH: Conceptualization, Funding acquisition, Resources, Supervision, Writing – review & editing. QL: Writing – original draft. HM: Data curation, Investigation, Writing – original draft. CM: Resources, Supervision, Writing – review & editing. JW: Resources, Supervision, Writing – review & editing. YM: Data curation, Investigation, Writing – review & editing. YZ: Data curation, Investigation, Writing – review & editing.

## Footnotes

^1^ https://www.aqistudy.cn/
