# Supplementary material for: Effects of the interaction between cold spells and fine particulate matter on mortality risk in Xining: a case-crossover study at high altitude
Source: Front Public Health. 2024 May 15;12:1414945. doi: 10.3389/fpubh.2024.1414945 (PMC11133570; doi:10.3389/fpubh.2024.1414945)
Supplement: Supplementary file 1 [file Data_Sheet_1.docx]

Supplementary Material

# Supplementary Tables

**Supplementary Table S1** Daily meteorological, pollutant and mortality data for Xining from 2016 to 2021.

| Variables | Counts | Mean ± SD | min | median | max |
| --- | --- | --- | --- | --- | --- |
| **Deaths** | | | | | |
| Non-accidental death | 64,128 | 29.26 ± 7.97 | 6 | 29 | 60 |
| Circulatory disease | 29,906 | 13.64 ± 4.87 | 1 | 13 | 34 |
| Respiratory disease | 8,553 | 3.93 ± 2.52 | 0 | 4 | 13 |
| IHD | 11,617 | 5.30 ± 2.66 | 0 | 5 | 19 |
| Stroke | 12,237 | 5.58 ± 2.74 | 0 | 5 | 19 |
| COPD | 7,074 | 3.10 ± 2.03 | 0 | 3 | 12 |
| Diabetes | 2,560 | 1.17 ± 1.16 | 0 | 1 | 7 |
| **Average altitude** | | | | | |
| 2500m | 33,179 | 15.14 ± 4.75 | 3 | 15 | 39 |
| 3000m | 30,888 | 14.09 ± 5.18 | 1 | 14 | 36 |
| **Sex** | | | | | |
| Male | 36,904 | 16.84 ± 5.22 | 2 | 17 | 38 |
| Female | 27,224 | 12.42 ± 4.36 | 1 | 12 | 32 |
| **Age** | | | | | |
| 0-64 years | 17,528 | 7.99 ± 3.12 | 0 | 8 | 23 |
| ≥65 years | 46,600 | 21.26 ± 6.62 | 4 | 21 | 48 |
| **Education Level** | | | | | |
| Low | 54,971 | 25.08 ± 7.38 | 5 | 24 | 53 |
| High | 9,157 | 4.18 ± 2.18 | 0 | 4 | 13 |
| **Meteorological variables** |  |  |  |  |  |
| Daily mean temperature (°C) | / | 6.46 ± 9.15 | -16.2 | 7.2 | 25.6 |
| Daily mean relative humidity (%) | / | 56.69 ± 16.20 | 15 | 57 | 94 |
| **Air pollutants** |  |  |  |  |  |
| PM2.5 (μg/m3) | / | 40.22 ± 27.90 | 4 | 33 | 392 |
| SO2 (μg/m3) | / | 20.03 ± 13.12 | 1 | 16 | 133 |
| NO2 (μg/m3) | / | 39.31 ± 16.11 | 1 | 36 | 110 |
| CO(mg/m3) | / | 1.36 ± 0.78 | 0.2 | 1.13 | 6.1 |
| O_3_(μg/m3) | / | 93.31 ± 33.47 | 11 | 92 | 281 |

**Supplementary Table S2** The relative excess odds due to interaction (REOI) of different cold wave definitions and PM_2.5_ on non-accidental deaths, categorised by altitude, gender, age and education level.

| Definitions | **Altitude** | | **Sex** | | **Age** | | **Educational level** | |
| --- | --- | --- | --- | --- | --- | --- | --- | --- |
|  | 2500 m | 3000 m | Male | Female | 0-64 years | ≥65 years | Low | High |
| 10th2D | 0.187 (0.073 to 0.301) | 0.091 (-0.026 to 0.207) | 0.141 (0.034 to 0.249 ) | 0.132 (0.007 to 0.258 ) | 0.057 (-0.097 to 0.211) | 0.168 (0.072 to 0.264) | 0.140 (0.052 to 0.227) | 0.121 (-0.105 to 0.347) |
| 10th3D | 0.203 (0.079 to 0.327) | 0.065 (-0.056 to 0.187) | 0.144 (0.031 to 0.258 ) | 0.119 (-0.015 to 0.254 ) | 0.010 (-0.156 to 0.176) | 0.179 (0.077 to 0.28) | 0.135 (0.042 to 0.229) | 0.114 (-0.120 to 0.348) |
| 10th4D | 0.204 (0.070 to 0.337) | 0.104 (-0.030 to 0.238) | 0.174 (0.052 to 0.296 ) | 0.127 (-0.022 to 0.275 ) | -0.043 (-0.232 to 0.146) | 0.224 (0.115 to 0.333) | 0.163 (0.062 to 0.265) | 0.083 (-0.171 to 0.338) |
| 5th2D | 0.271 (0.122 to 0.42) | 0.047 (-0.126 to 0.22) | 0.161 (0.015 to 0.308 ) | 0.170 (-0.007 to 0.348 ) | -0.024 (-0.248 to 0.200) | 0.232 (0.101 to 0.363) | 0.210 (0.089 to 0.331) | -0.108 (-0.421 to 0.206) |
| 5th3D | 0.272 (0.119 to 0.426) | 0.070 (-0.111 to 0.251) | 0.190 (0.037 to 0.342 ) | 0.160 (-0.022 to 0.343 ) | -0.046 (-0.281 to 0.189) | 0.256 (0.121 to 0.391) | 0.210 (0.086 to 0.334) | -0.035 (-0.376 to 0.306) |
| 5th4D | 0.362 (0.187 to 0.538) | 0.049 (-0.171 to 0.269) | 0.210 (0.026 to 0.394 ) | 0.228 (0.020 to 0.436 ) | -0.032 (-0.306 to 0.242) | 0.308 (0.149 to 0.468) | 0.251 (0.104 to 0.399) | 0.006 (-0.377 to 0.389) |
| 2.5th2D | 0.404 (0.213 to 0.595) | 0.122 (-0.092 to 0.337) | 0.198 (0.002 to 0.395 ) | 0.354 (0.148 to 0.56 ) | 0.018 (-0.255 to 0.291) | 0.360 (0.194 to 0.527) | 0.308 (0.158 to 0.457) | 0.008 (-0.423 to 0.439) |
| 2.5th3D | 0.435 (0.224 to 0.645) | 0.102 (-0.111 to 0.315) | 0.194 (-0.011 to 0.4 ) | 0.354 (0.138 to 0.571 ) | 0.021 (-0.257 to 0.299) | 0.361 (0.185 to 0.537) | 0.301 (0.145 to 0.457) | 0.038 (-0.423 to 0.498) |
| 2.5th4D | 0.369 (0.128 to 0.609) | -0.015 (-0.287 to 0.257) | 0.121 (-0.126 to 0.367 ) | 0.256 (-0.005 to 0.516 ) | -0.061 (-0.404 to 0.281) | 0.272 (0.062 to 0.483) | 0.201 (0.014 to 0.389) | 0.036 (-0.535 to 0.606) |

**Supplementary Table S3** The proportion attributable to interaction (AP) of different cold wave definitions and PM_2.5_ on non-accidental deaths, categorised by altitude, gender, age and education level.

| Definitions | **Altitude** | | **Sex** | | **Age** | | **Educational level** | |
| --- | --- | --- | --- | --- | --- | --- | --- | --- |
|  | 2500 m | 3000 m | Male | Female | 0-64 years | ≥65 years | Low | High |
| 10th2D | 0.172 (0.152 to 0.192) | 0.086 (0.076 to 0.097) | 0.136 (0.120 to 0.152) | 0.120 (0.104 to 0.135) | 0.059 (0.048 to 0.069) | 0.153 (0.138 to 0.168) | 0.131 (0.119 to 0.144) | 0.112 (0.085 to 0.138) |
| 10th3D | 0.179 (0.157 to 0.201) | 0.064 (0.055 to 0.072) | 0.138 (0.122 to 0.155) | 0.106 (0.092 to 0.12) | 0.011 (0.009 to 0.013) | 0.160 (0.144 to 0.176) | 0.126 (0.113 to 0.138) | 0.108 (0.081 to 0.135) |
| 10th4D | 0.189 (0.162 to 0.215) | 0.102 (0.087 to 0.118) | 0.173 (0.149 to 0.197) | 0.114 (0.097 to 0.132) | -0.046 (-0.057 to -0.035) | 0.206 (0.182 to 0.229) | 0.155 (0.138 to 0.172) | 0.085 (0.059 to 0.111) |
| 5th2D | 0.257 (0.221 to 0.294) | 0.045 (0.037 to 0.052) | 0.160 (0.136 to 0.183) | 0.154 (0.129 to 0.179) | -0.025 (-0.032 to -0.019) | 0.213 (0.187 to 0.239) | 0.196 (0.174 to 0.218) | -0.120 (-0.164 to -0.075) |
| 5th3D | 0.263 (0.224 to 0.302) | 0.066 (0.054 to 0.078) | 0.186 (0.157 to 0.214) | 0.148 (0.122 to 0.173) | -0.05 (-0.063 to -0.036) | 0.235 (0.205 to 0.264) | 0.199 (0.175 to 0.223) | -0.035 (-0.048 to -0.022) |
| 5th4D | 0.327 (0.277 to 0.377) | 0.043 (0.035 to 0.052) | 0.191 (0.159 to 0.224) | 0.200 (0.164 to 0.236) | -0.033 (-0.043 to -0.023) | 0.264 (0.228 to 0.3) | 0.222 (0.193 to 0.251) | 0.006 (0.004 to 0.009) |
| 2.5th2D | 0.363 (0.309 to 0.418) | 0.116 (0.093 to 0.139) | 0.184 (0.151 to 0.216) | 0.324 (0.270 to 0.378) | 0.02 (0.014 to 0.026) | 0.314 (0.273 to 0.355) | 0.284 (0.248 to 0.32) | 0.008 (0.005 to 0.011) |
| 2.5th3D | 0.370 (0.315 to 0.425) | 0.101 (0.081 to 0.121) | 0.179 (0.147 to 0.210) | 0.323 (0.269 to 0.376) | 0.023 (0.016 to 0.03) | 0.311 (0.270 to 0.352) | 0.278 (0.243 to 0.314) | 0.033 (0.020 to 0.046) |
| 2.5th4D | 0.319 (0.261 to 0.378) | -0.014 (-0.018 to -0.01) | 0.110 (0.086 to 0.134) | 0.228 (0.181 to 0.276) | -0.067 (-0.092 to -0.042) | 0.231 (0.193 to 0.269) | 0.184 (0.155 to 0.214) | 0.03 (0.016 to 0.043) |

**Supplementary Table S4** The synergy index (S) of different cold wave definitions and PM_2.5_ on non-accidental deaths, categorised by altitude, gender, age and education level.

| Definitions | **Altitude** | | **Sex** | | **Age** | | **Educational level** | |
| --- | --- | --- | --- | --- | --- | --- | --- | --- |
|  | 2500 m | 3000 m | Male | Female | 0-64 years | ≥65 years | Low | High |
| 10th2D | 1.207 (1.036 to 1.379) | 1.095 (0.943 to 1.246) | 1.157 (0.999 to 1.315) | 1.136 (0.973 to 1.299) | 1.063 (0.846 to 1.279) | 1.180 (1.046 to 1.314) | 1.151 (1.027 to 1.275) | 1.126 (0.836 to 1.416) |
| 10th3D | 1.218 (1.041 to 1.396) | 1.068 (0.911 to 1.226) | 1.161 (0.992 to 1.33) | 1.119 (0.955 to 1.282) | 1.011 (0.801 to 1.221) | 1.190 (1.048 to 1.333) | 1.144 (1.016 to 1.271) | 1.122 (0.808 to 1.435) |
| 10th4D | 1.233 (1.012 to 1.453) | 1.114 (0.917 to 1.310) | 1.210 (0.990 to 1.429) | 1.129 (0.935 to 1.323 ) | 0.956 (0.729 to 1.183) | 1.259 (1.071 to 1.447) | 1.183 (1.023 to 1.343) | 1.093 (0.713 to 1.473) |
| 5th2D | 1.347 (1.048 to 1.646) | 1.047 (0.862 to 1.232) | 1.190 (0.959 to 1.420) | 1.182 (0.946 to 1.417) | 0.975 (0.727 to 1.224) | 1.270 (1.058 to 1.483) | 1.244 (1.051 to 1.436) | 0.893 (0.593 to 1.194) |
| 5th3D | 1.357 (1.031 to 1.683) | 1.071 (0.868 to 1.273) | 1.228 (0.970 to 1.485) | 1.173 (0.923 to 1.424) | 0.953 (0.696 to 1.210) | 1.307 (1.069 to 1.544) | 1.249 (1.040 to 1.457) | 0.966 (0.632 to 1.301) |
| 5th4D | 1.486 (1.077 to 1.894) | 1.045 (0.840 to 1.251) | 1.236 (0.965 to 1.508) | 1.250 (0.947 to 1.553) | 0.968 (0.683 to 1.253) | 1.358 (1.090 to 1.627) | 1.286 (1.055 to 1.516) | 1.006 (0.599 to 1.413) |
| 2.5th2D | 1.570 (1.099 to 2.041) | 1.131 (0.868 to 1.394) | 1.225 (0.947 to 1.502) | 1.479 (1.016 to 1.942) | 1.020 (0.672 to 1.368) | 1.458 (1.133 to 1.782) | 1.397 (1.102 to 1.692) | 1.008 (0.629 to 1.387) |
| 2.5th3D | 1.587 (1.132 to 2.041) | 1.112 (0.847 to 1.377) | 1.217 (0.945 to 1.490) | 1.477 (1.017 to 1.936) | 1.024 (0.672 to 1.375) | 1.452 (1.134 to 1.77) | 1.386 (1.095 to 1.677) | 1.034 (0.660 to 1.408) |
| 2.5th4D | 1.469 (1.008 to 1.931) | 0.986 (0.757 to 1.216) | 1.123 (0.857 to 1.390) | 1.296 (0.901 to 1.691) | 0.937 (0.597 to 1.278) | 1.301 (1.012 to 1.589) | 1.226 (0.968 to 1.484) | 1.030 (0.616 to 1.444) |

1. **Supplementary Figures**

**Supplementary Figure S1** Spearman correlation between air pollutants and meteorological factors in 2016-2020 of Xining City, Qinghai Province, China. (*P < 0.05).





**Supplementary Figure S2** The relationship between cold spells, PM_2.5_ and various causes of death (A and E are **IHD**,B and F are **Stroke**,C and G are **COPD**,D and H are **diabetes**). The horizontal black line indicates an OR of 1 and the vertical blue line indicates the threshold for high PM_2.5_ concentrations (>37.5 μg/m^3^). OR indicates ratio ratio; PM_2.5_ indicates fine particulate matter.


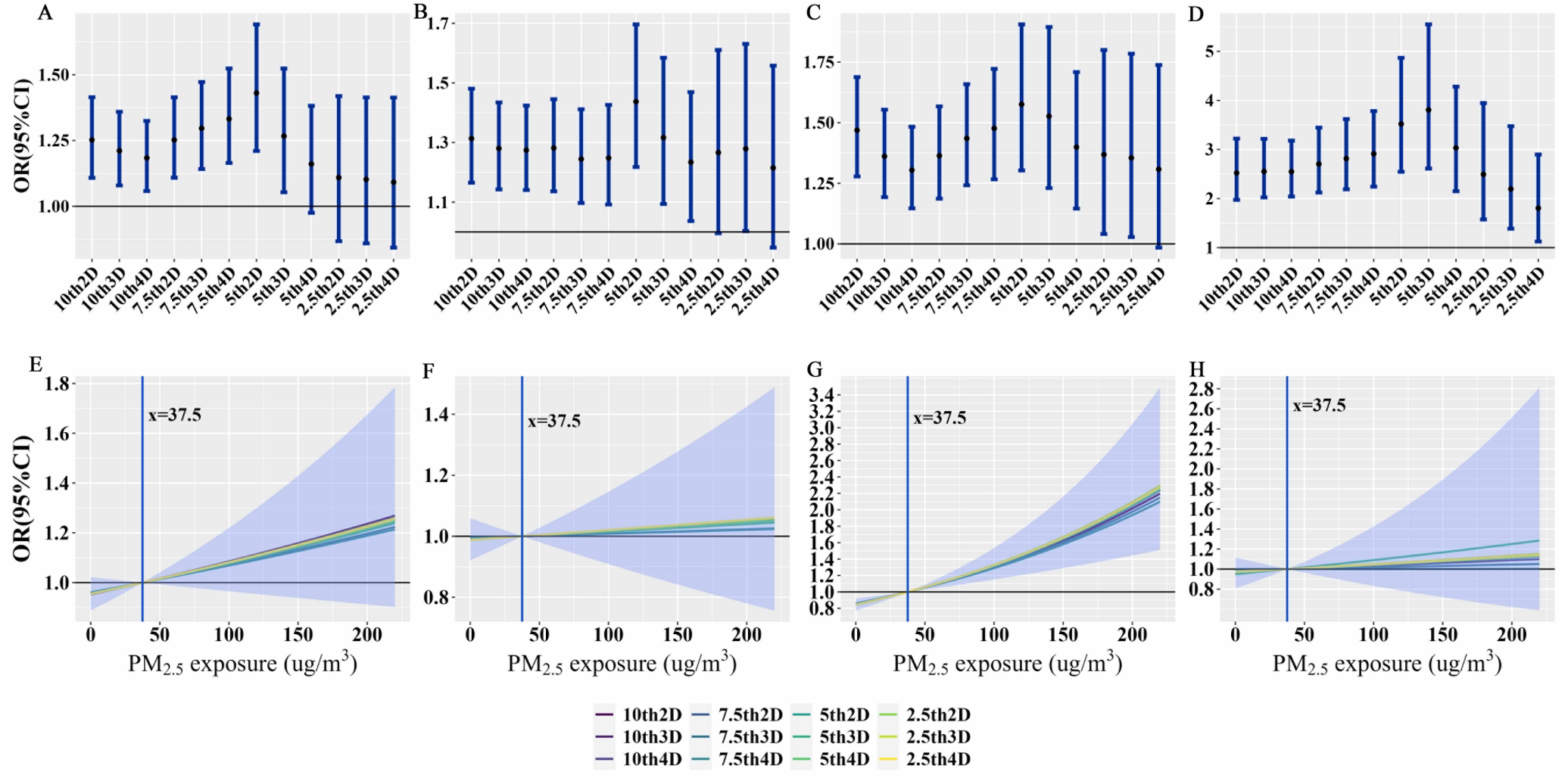


**Supplementary Figure S3** Additive interactive effects of exposure to cold spells and PM_2.5_ on specific causes of death (IHD, Stroke, COPD, and Diabetes).





**Supplementary Figure S4** Excess fraction due to exposure to cold spells and high levels of PM_2.5_ on specific causes of death (IHD, Stroke, COPD, and Diabetes).





**Supplementary Figure S5** Excess number due to exposure to cold spells and high levels of PM_2.5_ on specific causes of death (IHD, Stroke, COPD, and Diabetes).





**Supplementary Figure S6** The OR of cold spell on non-accidental deaths under different cold spell definitions by average altitude, sex, age and education level.





**Supplementary Figure S7** The OR of PM_2.5_ on non-accidental deaths under different cold spell definitions by average altitude, sex, age and education level.





**Supplementary Figure S8** The relative excess odds due to interaction (REOI) for sensitivity analyses of non-accidental deaths: adjusting the lag days from 0-21 to 0-27 for cold spells and from 0-7 to 0-10 for PM_2.5_, adjusting the degrees of freedom for relative humidity in the model, incorporating single air pollutants (SO_2_, NO_2_, CO, O_3_) as well as combined air pollutants (NO_2_&SO_2_&CO), and Use a PM_2.5_ classification value of 39.5.





**Supplementary Figure S9** The proportion attributable to interaction (AP) for sensitivity analyses of non-accidental deaths: adjusting the lag days from 0-21 to 0-27 for cold spells and from 0-7 to 0-10 for PM_2.5_, adjusting the degrees of freedom for relative humidity in the model, incorporating single air pollutants (SO_2_, NO_2_, CO, O_3_) as well as combined air pollutants (NO_2_&SO_2_&CO), and Use a PM_2.5_ classification value of 39.5.





**Supplementary Figure S10** The synergy index(S) for sensitivity analyses of non-accidental deaths: adjusting the lag days from 0-21 to 0-27 for cold spells and from 0-7 to 0-10 for PM_2.5_, adjusting the degrees of freedom for relative humidity in the model, incorporating single air pollutants (SO_2_, NO_2_, CO, O_3_) as well as combined air pollutants (NO_2_&SO_2_&CO), and Use a PM_2.5_ classification value of 39.5.





**Supplementary Figure S11** The ORs of cold spells for sensitivity analyses on non-accidental deaths: adjusting the lag days from 0-21 to 0-27 for cold spells and from 0-7 to 0-10 for PM_2.5_, adjusting the degrees of freedom for relative humidity in the model, incorporating single air pollutants (SO_2_, NO_2_, CO, O_3_) as well as combined air pollutants (NO_2_&SO_2_&CO), and Use a PM_2.5_ classification value of 39.5.





**Supplementary Figure S12** The ORs of PM_2.5_ for sensitivity analyses on non-accidental deaths: adjusting the lag days from 0-21 to 0-27 for cold spells and from 0-7 to 0-10 for PM_2.5_, adjusting the degrees of freedom for relative humidity in the model, incorporating single air pollutants (SO_2_, NO_2_, CO, O_3_) as well as combined air pollutants (NO_2_&SO_2_&CO), and Use a PM_2.5_ classification value of 39.5.





**Supplementary Figure S13** Separate effects of cold spells and PM_2.5_ before and after the 2019 coronavirus (COVID-19) outbreak.
